# Country adherence to WHO recommendations to improve the quality of HIV diagnosis: a global policy review

**DOI:** 10.1136/bmjgh-2019-001939

**Published:** 2020-05-05

**Authors:** Virginia A Fonner, Anita Sands, Carmen Figueroa, Rachel Baggaley, Caitlin Quinn, Muhammad S Jamil, Cheryl Johnson

**Affiliations:** 1 Department of Psychiatry and Behavioral Sciences, Medical University of South Carolina, Charleston, South Carolina, USA; 2 Department of Regulation and Prequalification, World Health Organization, Geneve, GE, Switzerland; 3 Global HIV, Hepatitis and STI Programme, World Health Organization, Geneva, GE, Switzerland

**Keywords:** diagnostics and tools, health policy, screening, HIV

## Abstract

**Introduction:**

Ensuring a correct and timely HIV diagnosis is critical. WHO publishes guidelines on HIV testing strategies that maximise the likelihood of correctly determining one’s HIV status. A review of national HIV testing policies in 2014 found low adherence to WHO guidelines. We updated this review to determine adherence to current recommendations.

**Methods:**

We conducted a comprehensive policy review through April 2018. We extracted data on HIV testing strategies, recommendations on HIV retesting prior to antiretroviral therapy (ART) initiation and pre-exposure prophylaxis (PrEP)-related HIV testing information. Descriptive analyses disaggregated by region were conducted to ascertain adherence to recommendations and to describe testing strategy characteristics.

**Results:**

Of 91 policies included, 26% (n=24/91) adhered to WHO recommendations. Having a two-assay testing strategy to rule-in HIV infection as opposed to the recommended three-assay testing strategy was a major reason for non-adherence. Of 72 country policies providing sufficient information, 31% (n=22) recommended retesting for HIV prior to initiating ART. Of 25 countries and two regions reporting PrEP-related HIV testing guidelines, almost all recommended testing prior to initiating PrEP and every 3 months during PrEP use.

**Conclusions:**

Global adherence to WHO recommendations for HIV testing strategies have improved since 2014 but remain low. We found adherence existed on a continuum. Such a system provides insights into how countries can move towards adherence by making relatively minor changes to testing strategies. Guidance from WHO on the role of new HIV testing technologies within testing algorithms and identifying ways to simplify testing guidance is warranted.

**Summary box**

**What is already known?**

- Receiving a correct diagnosis for HIV is critical for accessing treatment and prevention services. WHO publishes guidelines on HIV testing algorithms that maximise the likelihood of correctly determining one’s HIV status, yet global uptake of these recommendations is unknown.

**What are the new findings?**

- Global uptake of WHO recommendations for HIV testing services are low, with only 26% of country policies in adherence.

**What do the new findings imply?**

- Adherence exists on a continuum, and there are several small steps countries could take that would greatly increase adherence and minimise the likelihood of an incorrect diagnosis.
- More guidance from WHO is needed on new HIV testing technology and ways to simplify testing guidance.

## Introduction

HIV testing services (HTS) are the critical gateway to accessing HIV-related care and treatment for those diagnosed as HIV positive and as a means to accessing prevention services for those testing HIV negative. Despite its importance and recent testing scale-up to reach the ‘90-90-90’ targets set by UNAIDS, an estimated 21% of people living with HIV remain unaware of their serostatus.^1^

The HIV testing, treatment and prevention landscape has recently undergone rapid change. For example, in 2015, WHO recommended initiating antiretroviral therapy (ART) for all individuals living with HIV immediately on receiving an HIV-positive diagnosis, regardless of CD4 cell count (‘test and treat’).^2^ WHO also recommended the use of oral pre-exposure prophylaxis (PrEP) as an additional prevention option for individuals at substantial risk of HIV infection.^2^ Both recommendations bring new significance to HIV testing. With the test and treat approach, establishing correct HIV diagnoses becomes even more critical as an HIV-positive diagnosis becomes the sole criteria for initiating ART. Providing lifelong ART to someone misdiagnosed as HIV positive has substantial emotional, financial and psychosocial ramifications for the individual,^3–5^ as well as significant reputational and cost implications for programmes.^6^ Further, individuals initiating PrEP require HIV testing to confirm they are HIV negative and quarterly HIV testing during PrEP use.^7^ The WHO PrEP implementation tool suggests using the same HIV testing strategy, preferably the nationally verified testing algorithm, in the context of PrEP as recommended for HIV testing more generally.^7^

In recent years, in vitro diagnostic medical devices (IVDs) for detection of HIV have improved, making the diagnosis of HIV possible earlier in the course of infection.^8^ Quality-assured rapid diagnostic tests (RDTs) that detect antibodies to HIV are widely available, which enables HIV testing to be delivered at point of care. This same serological assay principle is also widely used in laboratory settings. Fourth generation HIV antibody/antigen RDTs and immunoassays (IAs) can potentially detect HIV during acute infection, when antibodies to HIV are not yet detectable. Nucleic acid testing (NAT) technologies are becoming more simplified and robust, and therefore more accessible in many settings. Nucleic acid is the first marker of HIV infection that may be detected. Currently, WHO does not have guidance relating to the suitability of NAT technologies for use in HIV testing strategies.

Despite these advances, uncertainty exists for any testing event as no single assay can provide a definitive HIV-positive diagnosis. Since 1997, WHO has recommended countries adopt one of two testing strategies depending on the HIV prevalence in the population undergoing testing—including one for low (<5%) and one for high (≥5%) prevalence settings. When adhered to, and populated with assays meeting 99% sensitivity and 98% specificity, these testing strategies give a positive predictive value of ≥99%.^9^ National guidelines outlining HIV testing strategies are critical to ensure HIV testing is carried out accurately and timely. However, a 2014 review of national HIV testing policies from 48 countries found that only 17% of testing strategies adhered to WHO guidelines.^10^

In 2015, WHO released consolidated guidelines on HTS.^11^ These guidelines maintained previous recommendations but added more guidance about HIV testing strategies and algorithms, including the order of assays to be performed based on sensitivity and specificity and an emphasis on national verification of testing algorithms. Additionally, the 2015 HTS guidelines highlighted the need to retest individuals newly diagnosed with HIV prior to starting ART, although this had previously been included in WHO guidance^12^ and information notes.^13^ The need for retesting prior to ART initiation was highlighted due to reports of misdiagnosis^14 15^ and the global shift towards test and treat^16^—making it imperative to provide a correct diagnosis before the initiation of life-long treatment. Recent mathematical modelling has shown retesting prior to ART initiation to be a cost-saving quality measure in high and low HIV prevalence settings.^6^

Given the millions of HIV tests conducted annually, having testing strategies in place to minimise misdiagnosis while maintaining quality is critical. Prior to 2014 and following the release of the 2015 HTS guidelines, WHO conducted dissemination events at regional and international conferences, provided technical support to countries and engaged partners and donors to support and promote implementation.

Considering the low uptake of WHO recommend testing strategies previously reported,^11^ we sought to update this review and to assess adherence to the 2015 WHO recommendations on HIV testing strategies and quality measures, including retesting prior to ART initiation. Additionally, in a subset of countries with guidance on HIV testing among PrEP users, we assessed the implementation of WHO recommended testing strategies among people initiating and/or taking PrEP.

## Methods

### Search strategy

We undertook a comprehensive search of national policy documents pertaining to HTS through searching WHO repositories, governmental and non-governmental websites, and contacting country and regional experts through April 2018 (online supplementary file 1). We included any HTS-relevant policy document, including national guidelines on HIV testing, strategic plans related to HIV, integrated guidelines on the prevention, treatment and care of HIV, and policies pertaining to PrEP. For inclusion, the policy must have reported on either a national HIV testing strategy and/or algorithm or an HIV testing strategy in the context of PrEP. There were no restrictions on language; however, policies in languages other than English had limited information extracted, with information on testing strategies prioritised. Date of policy publication was also not restricted. When policies for multiple years were identified for the same country, the most recent version containing relevant information on HTS strategies was included.

10.1136/bmjgh-2019-001939.supp1 Supplementary data

### Data extraction

Data were extracted from each policy by two independent reviewers into standardised coding forms (online supplementary file 2). Differences between coders were resolved through consensus. To prevent misclassification, items were marked as ‘unclear’ during data extraction when lack of translation and/or lack of information prevented complete understanding. Data were extracted within the following categories: (1) policy information (publication year, location, HIV prevalence); (2) HTS strategy (serial/parallel, number of assays, use of tie breaker to rule in HIV infection, mention of sensitivity and specificity in selecting assay order; use of WHO prequalified IVDs; assay names and types, sensitivity/specificity, mention of in-country verification; alignment or misalignment of testing strategy with WHO recommendations; (3) retesting before ART initiation; and (4) HIV testing in the context of PrEP (testing strategies/algorithms, frequency and description of testing).

10.1136/bmjgh-2019-001939.supp2 Supplementary data

### Analysis

Adherence to WHO recommendations was assessed using criteria set forth in the 2012 WHO Guidance, ‘Service delivery approaches to HIV testing and counselling (HTC): A strategic policy framework’^17^ and expanded on in the 2015 WHO Guidance, ‘Consolidated guidelines on HIV testing services’ (box 1).^11^

Box 1 **WHO guidelines for HIV testing strategies**

**WHO guidelines contain the following principles for HIV testing strategies:**

- National testing strategies should conform to either the high (≥5%) or low (<5%) prevalence HIV testing strategy according to UNAIDS data on prevalence.^130^
- Testing specimens in a serial manner, meaning that the result of assay 1 is read and interpreted before deciding to proceed to assay 2, and so on.
- Assay 3 should not be used as a tie breaker to rule in HIV infection. But assay 3 may be used across all settings to rule-out HIV infection.
- Each assay in the testing algorithm should have a sensitivity of ≥99%, while assay 1 should have ≥98% specificity and assay 2 and assay 3≥99% specificity.
- Testing algorithms should be verified locally (eg, national or regional).
- Retest all people newly diagnosed with HIV, using the national verified testing algorithm, prior to ART initiation

**Specifically for high prevalence settings (≥5%):**

- Specimens reactive on assay 1 should be tested on another assay (assay 2).
- If the specimen is also reactive on assay 2, result should be reported as HIV positive.
- If results between assay 1 and assay 2 are discrepant, both assays should be repeated.
- On repeat, if test results remain discrepant, assay 3 should be conducted.
  - If assay 3 is non-reactive (A1+, A2−, A3−), the result should be reported as HIV negative.
  - If assay 3 is HIV reactive (A1+, A2−, A3+), the result should be reported as HIV inconclusive and the individual should be retested after 14 days.

**Specifically for low-prevalence countries (<5%):**

- Specimens reactive on assay 1 should be tested on another assay (assay 2).
- Then, specimens reactive on assay 2 should be tested on another assay (assay 3).
- Specimens reactive on assay 3 should be reported as HIV positive.
- If results between assay 1 and assay 2 are discrepant (A1+, A2−), repeat both assays.
  - After repeat, if the assays are both non-reactive (A1−, A2−), the result should be reported as HIV negative.
  - After repeat, if the test results are still discrepant, the individual should be reported as HIV negative when assay 1 and assay 2 are either second or third generation assay principles. However, if assay 1 is fourth generation assay, the individual should be reported as HIV inconclusive and retesting recommended after 14 days.
- After repeat, if assay 1 and assay 2 are both reactive (A1+, A2+), the specimen should be tested with assay 3. If assay 3 is reactive, the result should be reported as HIV positive. If assay 3 is non-reactive (A1+, A2+, A3−), the result should be reported as HIV inconclusive and retesting should be performed after 14 days.

Descriptive analyses, disaggregated by WHO region, were conducted to determine rates of adherence to WHO recommendations; use of serial or parallel testing strategies; number, type, brand and prequalification status of assays used, and use of the assay 3 test result as a tie breaker to rule-in HIV infection. Analyses were conducted in Microsoft Excel and data were visualised using Tableau V.2018.

### Categorisation

Each testing strategy was assessed for adherence to WHO guidance using the information available. When assigning adherence, reviewers assessed alignment with WHO criteria and listed primary reasons for misalignment. Primary reasons for non-adherence were qualitatively assessed and divided into minor and major issues (Box 2). The major and minor categories were determined by consensus based on the severity of issues as they pertain to the potential for misdiagnosis, as well as cost and quality concerns. For example, parallel testing was classified as a ‘major issue’ because it is costlier than serial testing and leads to a greater number of discrepant results, thus contributing to other quality issues and further resource increases.

Box 2 **Minor and major issues with HIV testing strategy/algorithm non-adherence**

**Minor issues of non-adherence**

- Performing unnecessary Assay 3 following repeated discrepant Assay 1/Assay 2 results in low prevalence settings (should have been ruled HIV-negative after second round of discrepant Assay 1/Assay 2, or HIV-inconclusive if Assay 1 was 4th generation assay)
- Not repeating Assay 1/Assay 2 at all upon discrepant results, or not repeating Assay 1/Assay 2 immediately upon discrepant results (e.g., waiting 14 days instead)
- Not mentioning a specific Assay 3 but alluding to an additional assay performed at a laboratory
- Not using WHO prequalified diagnostics in testing algorithm

**Major issues of non-adherenc**

- Not having Assay 3 to resolve discrepant A1/A2 in high prevalence settings
- Using a parallel testing strategy
- Reporting an HIV-positive diagnosis based on reactive test results from only two assays in a low prevalence setting
- Using the result of Assay 3 as a tie-breaker to rule-in HIV infection

**Table 1:** HIV testing strategy characteristics by WHO region

WHO regional office for	Adherence to WHO rec, n (%)	Use of tie breaker, N (%)	Retesting prior to ART, N (%)
**Africa (n=32**)	9 (28%)	6 (19%)	16 (50%)
**Eastern Mediterranean (n=11**)	5 (45%)	2 (18%)	4 (35%)
**Europe (n=14**)	3 (21%)	0 (0%)	0 (0%)
**Americans and the Caribbean (n=16**)	1 (6%)	5 (16%)	1 (6%)
**Southeast Asia (n=8**)	5 (63%)	1 (13%)	1 (13%)
**Western Pacific (n=10**)	1 (10%)	0 (0%)	0 (0%)

Categorisation of testing strategies included:

- Fully adherent: no minor or major issues identified.
- Mostly adherent: one minor issue identified.
- Somewhat not adherent: two or more minor issues identified.
- Not adherent: one or more major issues identified.

To determine the proportion of countries adherent to WHO recommendations for HIV testing, country policies categorised as ‘full adherent’ and ‘mostly adherent’ were considered adherent and those categorised as ‘somewhat not adherent’ or ‘not adherent’ were considered non-adherent.

## Results

National policy documents related to HTS were identified for 146 countries. Of these, 55 were excluded from analysis for (1) not containing specific information on HIV testing strategies (eg, no details on order of assays, assays used, or algorithm (n=34)) and (2) an inability to extract information due to translation issues (n=21). Of the remaining 91 policy documents, each contained at least one piece of required information, but only 76 provided enough information to assess alignment with WHO HIV testing strategies. Twenty-seven policy documents contained information on PrEP-related HIV testing.

**Figure 1:**
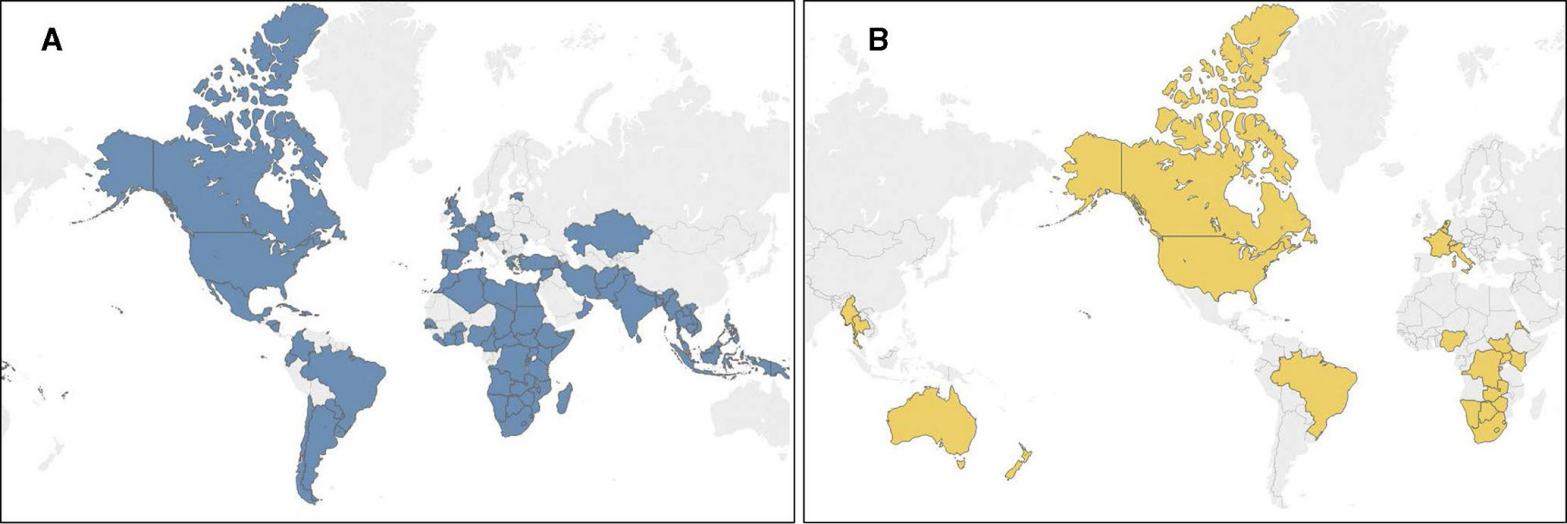
(A) Countries with HIV testing services policies identified and included in analysis (n=91). (B) Countries with policies identified containing information on HIV testing in the context of pre-exposure prophylaxis (n=27).

Of 91 countries providing data on HIV testing strategies,^18–108^ 32 were from the Africa region (AFRO), 11 from Eastern Mediterranean region (EMRO), 14 from the European region (EURO), 16 from the region of the Americas (AMRO), 8 from the South-East Asia region (SEARO) and 10 from the Western Pacific region (WPRO; figure 1a). Countries contributing data accounted for the vast majority of Fast Track Countries identified by UNAIDS (n=26/30), which comprise 89% of new HIV infections globally.^109^

Regarding HIV testing in the context of PrEP, 25 country policies^21 22 43 45 51 68 72 75 84 88 90 95 99 110–120^ and 2 regional policies^121 122^ (from the European Union and Australia/New Zealand) were included, of which 14 were from AFRO, 6 from EURO, 4 from AMRO, 2 from SEARO, and 1 from the WPRO (figure 1b).

Policy publication dates ranged from 2005 and 2018. Approximately 75% of included policies were published from 2013 to 2018. All policies related to HIV testing in the context of PrEP were published from 2016 onwards.

### Adherence to HIV testing strategies

Overall, 26% (n=24/91) of national testing strategies were either adherent or mostly adherent to WHO recommendations, and 57% (n=52/91) of strategies were non-adherent. The remaining 16% (n=15/91) did not contain enough information to determine adherence. In high HIV prevalence countries, 50% of policies (n=6/12) were adherent or mostly adherent, and the most common major reason for non-adherence was lack of an assay 3 to resolve discrepant assay 1 and assay 2 results.

In low-prevalence countries (n=79), 23% of national strategies (n=18/79) were adherent or mostly adherent, 59% (n=47/79) were non-adherent and 18% (n=14/79) did not contain enough information to determine adherence. Among low-prevalence countries, the most common major reason for non-adherence was diagnosing HIV based on reactive results for only two assays (n=35/47).

**Figure 2:**
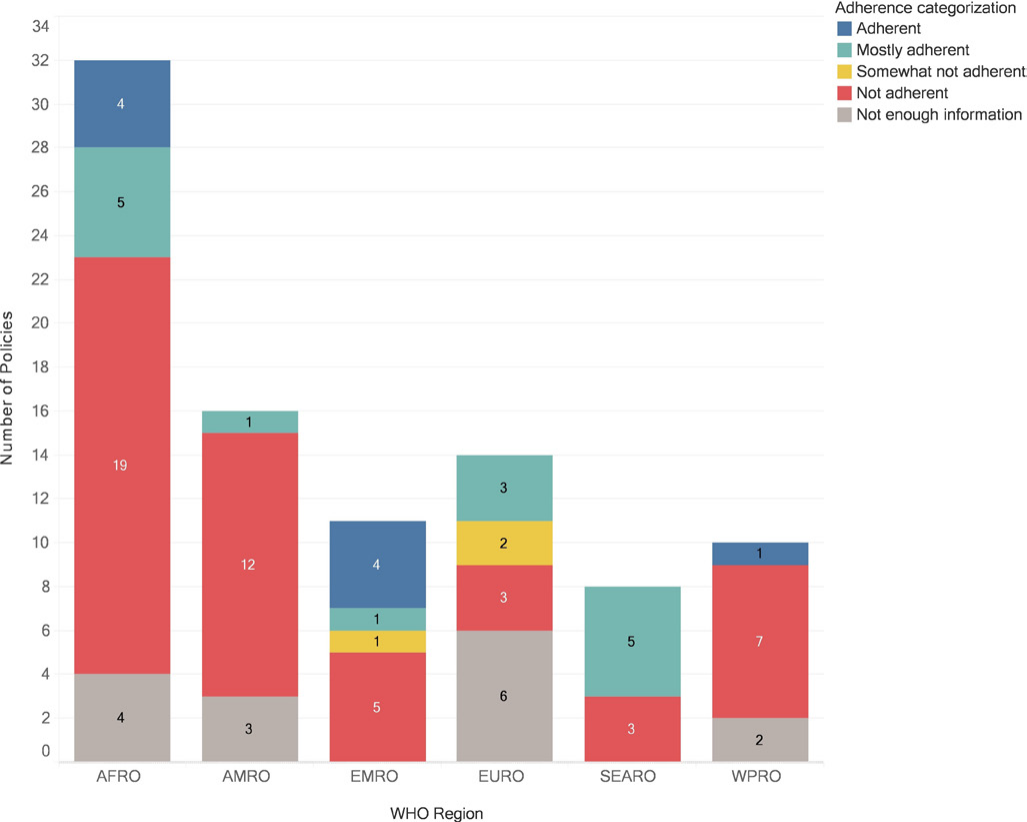
Adherence to WHO recommendations for HIV testing strategy by region. Adherence to WHO testing recommendations for each WHO region. Colour shows details about compliance categorisation (full adherence=no major or minor issues; mostly adherent=one minor issues; somewhat not adherent=two or more minor issues; not adherent=one or more major issues).

When stratified by WHO region, SEARO had the highest rate of adherence (n=5/8, 63%), followed by EMRO (n=5/11, 45%) and AFRO (n=9/32, 28%; table 1; figure 2). AMRO had the lowest rate of compliance (n=1/16, 6%), mostly due to testing strategies that reported HIV-positive diagnosis based on reactive results for only two assays. Online supplementary file 3 contains adherence information for all national policies contributing data.

10.1136/bmjgh-2019-001939.supp3 Supplementary data

When restricted to the subset of countries with policies included in the 2014 review that also had an updated policy available for the current review (n=21), the proportion adhering to WHO guidelines in 2014 was 9.5% (n=2/21) compared with 38% in the current review (n=8/21). The majority of countries moving from non-adherence to adherence from 2014 to the present came from sub-Saharan Africa (n=5/7).

### Characteristics of testing strategies and testing algorithms

Of included policies, 84% (n=76/91) used a serial testing strategy, 10% (n=9/91) used a testing strategy involving some element of parallel testing, and in 7% (n=6/91) it was unclear whether a serial or parallel testing strategy was used. Twenty-seven per cent of strategies (n=25/91) contained two assays, 58% contained three assays (n=53/91), 6% contained more than three assays (n=6/91) and in 8% (n=7/91) the number of assays used was unclear. In some cases, a third assay was not specified but was alluded to in referring specimens to a laboratory for further testing, although often the specifics of such referrals were unclear. For testing strategies containing more than three assays, often this occurred when testing strategies recommended that specimens be sent to a laboratory after performing two assays, with multiple assays recommended on reaching the reference laboratory.

Few policies contained the specific testing algorithms used in testing strategies. Only 32% of policy documents (n=29/91) mentioned specific product names. Sixteen policies mentioned the need to verify the testing algorithm or specified that laboratories should verify the algorithm, and only one national testing policy provided outcomes of the testing algorithm verification study itself. Of 29 policies including information on assays (ie, specific product names), all but one included WHO prequalified products.

Seventy per cent (n=64/91) of policies provided the assay types used, for example, RDT, IA and western blotting. Of 64 policies reporting assay types, RDTs and IAs were most common. Thirty-one per cent (n=20/64) included western blotting as an option for either assay 2 or assay 3 (most commonly assay 3) and 13% (n=8/64) included NAT as an option for assay 2 or assay 3. Use of western blotting was common in EURO, EMRO and AMRO. Several high-income and middle-income countries reported using NAT technologies.

Overall, 15% (n=14/91) of testing strategies used results of assay 3 as a tie breaker to rule-in HIV infection (table 1); however, three specified the use of western blot or NAT as assay 3 to rule-in HIV infection. Most cases of using assay 3 as a tie breaker to rule-in HIV infection occurred in AFRO, mostly from West and North Africa (50%, n=7/14), and AMRO (36%, n=5/14). Of note, several countries referred to assay 3 as a ‘tie breaker’ although it was used correctly, that is, to rule-out HIV infection.

### Other issues with HIV testing strategies

Several policies specified different testing strategies for the purposes of diagnosis and surveillance, which contradicts WHO recommendations to use the same testing strategies for both purposes. Additionally, some policies contained testing strategies where the number of assays varied depending on certain circumstances, such as the availability of assay 3 and whether signs and symptoms of HIV infection are present. Several low-prevalence countries included a high-prevalence testing strategy when working with key populations and a separate low-prevalence testing strategy for the general population.

### Retesting prior to art initiation

Of 91 policies, 72 provided information on HIV retesting prior to starting ART (19 were excluded due to translation issues or for not having enough information). Of 72 policies, 31% (n=22) required or recommended retesting prior to ART initiation, and 86% of these (19/22) were published after 2015. The majority of policies requiring retesting were from AFRO (73%, n=16/22). The remaining 49 policies either did not require retesting prior to ART or did not specify whether retesting was required. Some policies required only written proof of an HIV diagnosis prior to initiating ART and only recommended retesting if written confirmation was unavailable. Two national strategies required retesting but used a parallel testing strategy as opposed to a serial testing strategy for retesting, which differed from national policy. Other countries specified that retesting should be performed with a different specimen and a different testing provider, in alignment with WHO recommendations.

### HIV testing in the context of PreP

Twenty-five national policies and two regional policies (from the European Union and Australia/New Zealand) contained information regarding HIV testing in the context of PrEP. Of 27 policies, almost all (n=24/27) recommended HIV testing occur at PrEP initiation and every 3 months while taking PrEP. The remaining three policies did not specify a schedule for HIV testing in the context of PrEP. Several policies additionally recommended retesting for HIV after 1 month of PrEP use and then 3 months thereafter. Two policies did not specify a testing schedule for PrEP users. Only four policies explicitly stated that HIV testing in the context of PrEP should follow the national verified HIV testing algorithm. Other policies did not specify what testing strategy or algorithm should be used, and one policy outlined a testing strategy for PrEP initiators and PrEP users that differed from the national testing strategy. Specifically, in this policy if a potential PrEP user had a non-reactive fourth generation antibody/antigen assay but reported symptoms of acute infection, NAT testing was recommended. Several other countries emphasised the need to assess signs and symptoms of acute HIV infection prior to initiating PrEP, and if present, to defer PrEP initiation until reviewing the result of further testing. Many high-income countries stipulated the use of fourth generation antigen/antibody assay for PrEP users and cautioned against the use of oral fluid-based serological assays. Policies from several countries included specific information on PrEP in their ARV guidelines, but clarified that PrEP was still under consideration for use in the country and therefore not yet available. Online supplementary file 3 contains data on policies providing information on HIV testing in the context of PrEP.

## Discussion

This review found that slightly over a quarter of countries are fully or mostly adherent to WHO HIV testing recommendations and that one-third recommend retesting for HIV prior to ART initiation. We found improvements in national HIV testing policies’ adherence to WHO guidelines as compared with a global review conducted in 2014.^10^ Specifically, rates of using the result of assay 3 as a tie breaker to rule-in HIV infection have declined. Additionally, a considerable proportion of HIV testing policies published after the 2015 WHO HTS recommendations adopted policy changes to maintain alignment with global guidelines, including adding requirements to retest newly diagnosed HIV-positive individuals for HIV prior to ART initiation and to regularly test PrEP users for HIV in a schedule adherent to WHO recommendations.

Overall adherence remains low. High-prevalence countries had proportionally higher rates of adherence than low-prevalence countries. Notably, of 12 strategies for high-prevalence countries identified, several have recently fallen below the 5% prevalence threshold.^1^ As HIV prevalence continues to decline globally, countries currently using a high-prevalence strategy may need to switch to a low-prevalence strategy, which WHO recently began encouraging countries to do.^123 124^ Eventually moving all countries to a low-prevalence testing strategy requiring reactive results from three assays to confirm HIV infection might be needed. Additionally, several countries noted that while the national HIV prevalence is below 5%, prevalence among key populations (eg, sex workers, men who have sex with men) is high (>5%), and thus policies suggested using a high-prevalence strategy among key populations. However, in these cases, it is unclear how the testing facility and/or tester would be aware of someone’s membership in a key population that would warrant using a different testing strategy.

We found that determination of adherence was often not a clear distinction, and adherence was more accurately defined using a continuum with several levels of gradation. Many policies contained strategies that were close to adhering but included minor deviations. Conversely, other countries outlined testing strategies that clearly contradicted WHO recommendations. As a result, a more nuanced categorisation scheme was developed. Using the continuum of adherence framework, making relatively minor changes to many strategies could greatly improve alignment.

There are several adaptations countries could undertake within national testing strategies to improve adherence. First and foremost, using IVDs that have been stringently assessed (such as by a founding member of the Global Harmonization Task Force or that are WHO prequalified) ensures the testing algorithm contains assays that have been assessed for quality, safety, and performance. Second, verifying the testing algorithm to ensure that assays do not share false reactive results would improve testing quality. A recent study assessing the performance of different testing algorithms across six sites in sub-Saharan Africa found suboptimal performance of multiple RDTs with site-specific differences, thus highlighting the critical importance of verifying algorithms.^125^

We found that approximately 30% of HIV testing strategies used western blot and over 10% used NAT in testing algorithms. WHO recently released guidance encouraging countries to move away from using western blotting and line IAs in HIV testing algorithms in favour of simpler tests.^124 126^ Further guidance regarding the use of NAT in testing algorithms would be beneficial. For example, undetectable HIV viral load should not be used to rule out HIV infection as it is possible to be HIV positive with undetectable HIV viral load. There is a theoretical possibility that NAT may be used to rule in HIV infection, but clinical utility studies to support this intended use are ongoing. Regarding HIV testing in the context of PrEP, the WHO PrEP implementation tool notes that using assays capable of identifying acute infection, such as NAT, where feasible may offer additional benefits as those seeking PrEP could be at heightened risk of having acute HIV infection.^7^ However, to date, WHO has not issued recommendations on the use of NAT in the context of PrEP.

The majority of non-adherence issues for low-prevalence settings centred on diagnosing HIV infection using only two assays. This deviation has the potential to increase the occurrence of misdiagnosis, particularly false-positive diagnosis. According to previous modelling by WHO, using reactive test results from two assays in low-prevalence settings would lead to reduced positive predictive value as low as 90.8% in settings where prevalence is 0.1%,^9^ well below the 99% positive predictive value recommended. In a population of one million people who are unaware of their HIV status, this could cause up to 100 individuals to be placed on ART who are HIV negative.

While correct use of assay 3 results was poor in low HIV prevalence settings, many policies included strategies that used assay 3 to resolve discrepant test results from the first two assays. Thus, changing the testing strategy so that an assay 3 is used to confirm HIV infection for individuals who are reactive on the first two assays may be feasible. Implementing this shift across settings would increase adherence with WHO recommendations from 26% to approximately 50%.

For high-prevalence settings, the primary issue was not having an assay three to resolve discrepant results from the first two assays. While misdiagnosis in these scenarios is not of primary concern, lacking an assay 3 to resolve discrepancies leads to important missed opportunities to provide a definitive HIV-negative diagnosis to those at ongoing risk for HIV and offer PrEP if warranted. Adding an assay 3 to the testing strategy or putting in clear referral mechanisms to a laboratory that could conduct an assay 3 is needed. For countries using an assay 3 inappropriately to rule in HIV infection, strategies could be altered to allow assay 3 to rule out HIV infection only.

Minor issues identified in strategies, such as unnecessarily using an assay 3 to resolve discrepant results from assay 1 and assay 2 or not repeating the first and second assays in case of discrepant results, if corrected, could improve efficiency and cost related to HIV testing.

Given the relatively low rates of adherence to WHO recommendations found in this review, more understanding of why countries choose or choose not to adopt WHO HIV testing recommendations is needed. Given the unique context, resources and processes that guide national policy formation, having more pragmatic data on these critical aspects could help inform future guideline development. Although we did not collect such data, our results highlight specific areas of potential inquiry, such as investigating why so many countries fail to use a third assay in their national testing strategies. A study of country adaptation of WHO guidelines for the prevention of mother-to-child transmission of HIV found that factors such as perceived complexity, cost, equity and existing healthcare infrastructure all factored into policy decision-making.^127^ Such a study on countries’ development of HIV testing strategies is warranted. Research is also needed to understand the effectiveness of WHO’s strategies for dissemination of HTS guidelines as it is possible that countries are unaware of the most recent changes to global recommendations.

The HIV testing strategies recommended by WHO require certain costs and resources. The overall perceived cost may be a barrier to widespread implementation of standardised HIV testing strategies. It is for this reason that WHO recommends a differentiated HTS delivery approach, including community-based testing via a ‘test for triage’ and HIV self-testing, which work to expand access to HIV testing while not compromising the need to have the full testing strategy available at all testing sites. Both a ‘test for triage’ and HIV self-testing offer a single RDT administered in a community setting with linkage available to facility-based testing for the confirmation of HIV diagnoses and to clinical care when needed.^128 129^

### Strengths and limitations

This review identified HTS policy documents from over 140 countries and analysed policies from over 90 countries. The review used robust searching methods and standardised, systemic data extraction. Despite the study team’s comprehensive efforts, it is possible that existing policy documents were not identified as only policies that were publicly available or received through government contacts and country offices via WHO were included. Additionally, because of the nature of the documents studied, this review used a non-automatized search strategy; nonetheless, we used keywords when possible, making the search strategy defined and repeatable.

Additionally, we did not ascertain country implementation of HTS policies. Countries might be implementing HIV testing strategies different from the ones outlined in official policy documents, thus having a non-adherent testing strategy as written policy with adherent implementation or vice versa. Although we did not include subnational policy documents, it is possible that decentralisation has led to variations in testing strategies at the subnational level that were missed by this review. Few countries provided specific testing algorithms in national policy documents, which limited our ability to assess adherence with WHO recommendations on testing algorithms. Additionally, for several data collection points, including national/regional verification of testing algorithm, we were only able to assess whether a policy provided specific mention of a procedure for verification and not whether the verification actually occurred; it is possible procedures such as algorithm verification are occurring but are not being included in policy documents. Finally, the inability to translate all policy documents was a limitation; when possible, translations of testing strategies were prioritised.

## Conclusions

Recent changes to the HIV prevention and treatment landscape, including ‘test and treat’ and PrEP availability, have elevated the importance of receiving a correct HIV diagnosis—both HIV positive and HIV negative. Following WHO recommendations for HIV testing strategies, performing verification of testing algorithms, and using appropriate assays can ensure accurate HIV diagnosis in a cost-efficient and time-efficient manner. While this review found a significant proportion of countries not adhering to WHO recommendations, adherence existed on a continuum. Our findings suggest that many countries are moving in the direction of increasing adherence and these efforts must be supported and lead to implementation. It is imperative that this trend continues, and more countries adopt WHO recommendations to ensure quality HIV testing programmes, especially in regards to requiring HIV retesting prior to ART initiation and implementing HIV testing in accordance with national guidelines for PrEP users. Understanding reasons behind countries decisions to adopt or not adopt WHO recommendations could help identify potential solutions. Ways to simplify the WHO testing guidance are warranted, such as moving to one HIV testing strategy for all settings.
