# Supplementary material for: Country adherence to WHO recommendations to improve the quality of HIV diagnosis: a global policy review
Source: BMJ Glob Health. 2020 May 5;5(5):e001939. doi: 10.1136/bmjgh-2019-001939 (PMC7228476; doi:10.1136/bmjgh-2019-001939)
Supplement: Supplementary data [file bmjgh-2019-001939supp001.pdf]

Supplemental File 1: Search Strategy

The process for collecting HTS guidelines included the following methods:

1. Search of internal WHO database for HIV Testing Services (HTS) policy documents

2. Country by country search of AIDSFree HTS policy database (https://aidsfree.usaid.gov/resources/guidance-data/hts)

3. Country by country search of IAPAC/HIV Policy Watch website (http://www.hivpolicywatch.org/database.html)

4. Broad Google search for HTS policies from each WHO country using the following key words in English, French, Portuguese, and Spanish:

- country name AND “HIV testing” AND policy
- country name AND “HIV testing” AND guideline
- country name AND PrEP AND policy
- country name AND PrEP AND guideline
- country name AND “pre-exposure prophylaxis” AND policy
- country name AND “pre-exposure prophylaxis” AND guideline
