# Supplementary material for: Country adherence to WHO recommendations to improve the quality of HIV diagnosis: a global policy review
Source: BMJ Glob Health. 2020 May 5;5(5):e001939. doi: 10.1136/bmjgh-2019-001939 (PMC7228476; doi:10.1136/bmjgh-2019-001939)
Supplement: Supplementary data [file bmjgh-2019-001939supp002.pdf]

Data Extraction Form for WHO HTS Policy Review

1. Country and Policy Identification

Country	WHO Region	HIV Testing policy identified?	Year of policy publication	Type of policy doc	Document name (primary data source)	HIV prevalence (country-level)

2. HIV testing strategy

HIV testing strategy provided?	Image or text format?	Type of scenario in which algorithm is used	Number of assays used in algorithm	Serial or Parallel strategy	Use of tiebreaker	Mention of test order relating to sens/spec	high or low prev strategy used	Type of tests mentioned	Brand of tests mentioned	Are assays pre-qualified by WHO

A1 test name	A1 type of assay	A1 sens/spec	A2 test name	A2 type of assay	A2 sens/spec	A3 test name	A3 type of assay	A3 sens/spec	Source of assay sens/spec	Mention of in-country assay validation	Alignment with WHO rec?	Primary reason(s) algorithm fails to meet WHO rec	Other notes about testing strategy

3. Retesting prior to ART initiation

Retest required before starting ART?	Retest strategy same as national algorithm?	Notes about retesting strategy

4. HTS in the context of PrEP

Country	Region	Policy on PrEP identified	Document name	Year of publicatio n of PrEP policy	Does HIV testing for PrEP follow national algorithm?	Describe HIV testing strategy for PrEP users (if different from national algorithm)	Frequency of recommended testing?
