# Supplementary material for: Country adherence to WHO recommendations to improve the quality of HIV diagnosis: a global policy review
Source: BMJ Glob Health. 2020 May 5;5(5):e001939. doi: 10.1136/bmjgh-2019-001939 (PMC7228476; doi:10.1136/bmjgh-2019-001939)
Supplement: Supplementary data [file bmjgh-2019-001939supp003.pdf]

Supplementary File 3: Adherence analysis for HTS policies

a. National HTS Policies

Region	Country	Year of policy publication	Strategy In compliance?	Primary concerns with strategy	Retesting prior to ART initiation?
**AFRO**	Algeria	2013	In compliance	N/A	No
	Angola	2015	Not in compliance	Confirms HIV diagnosis with only 2 assays in low prevalence setting; Includes viral load as an option for a third test	No
	Botswana	2016	Mostly in compliance	Minor issue: Repeat testing in parallel for discrepant results and also for verification of status before ART initiation.	Yes
	Burkina Faso	2008	Not in compliance	Confirms HIV diagnosis with only 2 assays in low prevalence setting. A1 and A2 not repeated if discrepant	No
	Burundi	2016	Not in compliance	Confirms HIV+ diagnosis with only 2 assays in low prevalence setting; does not repeat A1/A2.	Yes
	Cameroon	2015	Not in compliance	Use of tie-breaker; confirms HIV diagnosis with only 2 assays in low prevalence setting;	No
	Central African Republic	2010	Not in compliance	Confirms HIV diagnosis with only 2 tests in low prevalence setting;	No
	Chad	2011	Not in compliance	Confirms HIV+ diagnosis with only 2 assays in low prevalence setting; referral to lab for indeterminate results.	No
	Comoros	2007	Not enough information	Unclear what happens for RDT reactive but EIA non-reactive. Description of a negative status infers that both the RDT and the EIA must be non-reactive.	Yes
	Cote d'Ivoire	2016	Not in compliance	Confirms HIV diagnosis with only 2 assays in low prevalence setting. Retesting to confirm status uses tiebreaker approach.	No
	Democratic Republic of Congo	2017	In compliance	N/A	Yes
	Ethiopia	2017	Not enough information	N/A	Yes
	Gambia	2014	Not in compliance	Confirms HIV+ diagnosis with only 2 assays in low prevalence setting	No
	Ghana	2014	Not in compliance	Confirms HIV diagnosis with only 2 assays in low prevalence setting.	No
	Kenya	2017	Mostly in compliance	The strategy itself includes two assays, but a note to refer indeterminate samples (after A1/A2 repeat) to lab for testing "using a separate and distinct assay from the screening and confirmatory tests," which is why it technically meets WHO standards	Yes
	Lesotho	2016	In compliance	N/A	Yes

Fonner VA, *et al. BMJ Global Health* 2020; 5:e001939. doi: 10.1136/bmjgh-2019-001939

	Liberia	2015	Not in compliance	A3 used as tiebreaker; A1 and A2 not repeated if discrepant; HIV+ diagnosis made with only 2 assays in low prevalence setting	No
	Madagascar	2011	Not enough information	Not enough information	Unclear
	Malawi	2016	Not in compliance	Only two assays; No A3 performed after repeated A1+A2-	Yes
	Mozambique	2016	Not in compliance	Only two assays; repeat of A1/A2 performed 3-4 weeks later if A1/A2 initially discordant; third test alluded to if still discordant at second visit	Unclear
	Namibia	2016	Not enough information	N/A	Yes
	Nigeria	2016	Not in compliance	Both serial or parallel strategies presented. Uses tie-breaker approach	Yes
	Rwanda	2016	Not in compliance	Confirms HIV diagnosis with only 2 assays in low prevalence setting	Yes
	Senegal	2017	Mostly in compliance	A3 is not WHO pre-qualified	Unclear
	Sierra Leone	2017	Not in compliance	Confirms HIV diagnosis with only 2 assays in low prevalence setting; use of tiebreaker to rule-in HIV Infection.	No
	South Africa	2016	Mostly in compliance	Lab-based testing requires two additional ELISAs (so technically 4 tests in algorithm)	Yes
	South Sudan	2017	Not in compliance	Confirms HIV diagnosis with only 2 tests in low prevalence setting; third assay used as tie-breaker to rule-in HIV infection	Yes
	Eswatini	2018	Not in compliance	Technically does not have A3 to resolve discrepant A1/A2 in high prevalence settings. Instead, client returns in 2 weeks. DNA PCR is A3.	Yes
	Uganda	2016	Mostly in compliance	No repeat of A1/A2 for initial discrepant results	No
	United Republic of Tanzania	2017	Not in compliance	No A3 for repeat discrepant A1 and A2 results in high prevalence setting	Yes
	Zambia	2018	Not in compliance	No A3 for repeat discordant A1 and A2 results in high prevalence setting	No
	Zimbabwe	2016	In compliance	N/A	Yes
**EMRO**	Afghanistan	2013	In compliance	N/A	Yes
	Egypt	2015	Somewhat not in compliance	If A2 is non-reactive but A1 was reactive, reports as negative; does not repeat A1/A2 if discrepant; uses WB as A3 to rule-in HIV infection	No
	Iran (Islamic Republic of)	2018	In compliance	N/A	No
	Libya	2010	Not in compliance	For A1+ A2-, does not repeat A1 and A2; only 2 assays overall	No
	Morocco	2010	Not in compliance	Confirms HIV diagnosis with only 2 assays in low prevalence setting; confirmation test is western blot	No
	Oman	2015	In compliance	N/A	Yes

Fonner VA, *et al. BMJ Global Health* 2020; 5:e001939. doi: 10.1136/bmjgh-2019-001939

	Pakistan	2017	Mostly in compliance	A1/A2 repeat occurs after 14 days	Yes
	Somalia	2017	Not in compliance	Does not repeat A1 and A2 for discrepant results on screening and confirmatory tests before reporting HIV-. Also, written text describes use of a tiebreaker.	No
	Sudan	2016	In compliance	N/A	Yes
	Syrian Arab Republic	2014	Not in compliance	No repeat of A1/A2 if discrepant (if discrepant, ruled HIV-) and uses WB as A3	unclear
	Tunisia	2014	Not in compliance	Confirms HIV+ diagnosis with two assays in low prevalence setting; does not repeat A1/A2 for rapid tests	No
**EURO**	Austria	2013	Not enough information	Algorithm reads like only 2 assays used for diagnosis. Using RDT followed by EIA or western blot (WB) confirmation, or EIA followed by WB confirmation.	No
	Belgium	2014	Not enough information	It is unclear how many assays are used	No
	Estonia	2012	Not enough information	Many algorithms are presented; some with more than 3 assays	No
	France	2009	Mostly in compliance	Does not repeat A2, only A1	unclear
	Germany	2016	Not in compliance	Confirms HIV+ diagnosis with two assays in low prevalence setting; also does not repeat A1/A2 if discrepant	Unclear
	Greece	2014	Not in compliance	Confirms HIV+ diagnosis with two assays in low prevalence setting; also does not repeat A1/A2 if discrepant	Unclear
	Israel	2013	Mostly in compliance	WB used as A3;final decision for A1+A2+A3- is negative (should be indeterminate)	Unclear
	Kazakhstan	2015	Mostly in compliance	Technically has 4 assays; 4th involves immunoblotting or immune-chromatographic test to detect proteins for HIV – ENV, GAG, PO	Unclear
	Republic of Moldova	2016	Somewhat not in compliance	Does not repeat A1 if A1/A2 are discrepant. If results are A1+/A2+/A3- (using NAT), algorithm proceeds to HIV-2 testing, instead of reported as HIV inconclusive with retesting after 14 days.	Unclear
	Montenegro	2005	Not enough information	N/A	No
	Portugal	2014	Not in compliance	Only 2 assays used for HIV+ diagnosis in low prevalence setting, although mentions sending sample to reference lab if positive; Repeats A1 (before A2 and does not repeat A2 in case of discrepant results); discrepant A1/A2 goes for RNA testing.	Unclear
	Spain	2014	Not enough information	Not enough information	No
	Turkey	2013	Somewhat not in compliance	A2 can be same/similar to A1; no repeat of A1/A2 upon discrepant results	Unclear
	United Kingdom of Great Britain	2016	Not enough information	Not enough information	No

Fonner VA, *et al. BMJ Global Health* 2020; 5:e001939. doi: 10.1136/bmjgh-2019-001939

	and Northern Ireland				
**AMRO**	Argentina	2015	Not in compliance	Doesn't provide 3rd assay on site. It seems to have a laboratory algorithm that aligns with WHO, it has 4th Gen EIA, followed by viral load or WB as A2 and then if WB is used then viral load could be used to confirm. Or just 4th Gen + WB or viral load would be considered HIV+.	No
	Brazil	2014	Not in compliance	The recommendation is following two reactive RDTs that clients received viral load to confirm diagnosis.	No
	Canada	2012	Not in compliance	High prevalence algorithm used in low prevalence setting; screening test repeated multiple times; text alludes to screening and confirmatory tests only (not 3 different assays)	No
	Chile	2009	Not enough information	N/A	Yes
	Colombia	2014	Not in compliance	High prevalence testing strategy used in low prevalence setting; result of third assay used at tie-breaker	No
	Cuba	2013	Not enough information	Only 2 assays in algorithm; no mention of how lab tests are performed	No
	Dominican Republic	2014	Not in compliance	A3 is used as a tie-breaker to rule-in HIV infection; also confirms HIV diagnosis with only two assays in low prevalence setting	No
	Ecuador	2011	Mostly in compliance	Does not repeat A1/A2 if discrepant; third assay(WB) used to rule-in HIV infection	No
	Haiti	2015	Not in compliance	Confirms HIV diagnosis with only 2 assays in low prevalence setting; No A3	Unclear
	Honduras	2013	Not in compliance	Confirms HIV+ diagnosis with only 2 assays in low prevalence setting	No
	Mexico	2011	Not enough information	Not enough information	N/A
	Nicaragua	2012	Not in compliance	For discordant A1 and A2, instead of repeating A1 and A2 uses A3 (ELISA); A1+A2- A3+ reported as HIV-positive	Unclear
	Paraguay	2012	Not in compliance	A1/A2 not repeated if discrepant; if discrepant, no A3 is run; If A1, A2 reactive, A3 (WB) decides positive or negative diagnosis	Unclear
	Trinidad and Tobago	2013	Not in compliance	Parallel testing and use of tiebreaker	No
	United States of America	2014	Not in compliance	Confirms HIV+ diagnosis with only two assays in low prevalence setting	No
	Uruguay	2006	Not in compliance	A2 and A3 run in parallel; A1 and A2 not repeated upon discrepant results; WB used as A3	No
**SEARO**	Bangladesh	2013	Mostly in compliance	Progression to A3 following discrepant repeat of A1/A2. And all RDT and EIA results confirmed with Western Blot. If two EIA	No

Fonner VA, *et al. BMJ Global Health* 2020; 5:e001939. doi: 10.1136/bmjgh-2019-001939

				results are discrepant, confirmation with Western blot.	
	India	2015	Mostly in compliance	Proceeds to A3 for double discrepant A1/A2; should be reported as negative	No
	Indonesia	2014	Mostly in compliance	Unnecessary 3rd assay for discordant A1/A2 repeat	No
	Maldives	2009	Not in compliance	Confirms HIV diagnosis with only 2 assays in low prevalence setting	No
	Myanmar	2017	Not in compliance	A1 and A2 performed in parallel	Yes
	Nepal	2014	Not in compliance	Confirms HIV+ diagnosis with two assays in low prevalence setting; 3rd test used as tie-breaker	No
	Sri Lanka	2016	Mostly in compliance	Unnecessary 3rd test for discordant A1/A2 repeat; uses WB as A3 (but used correctly)	No
	Thailand	2017	Mostly in compliance	Unnecessary 3rd test if A1/A2 discordant upon repeat	Unclear
**WPRO**	Cambodia	2012	In compliance	N/A	No
	Fiji	2013	Not in compliance	Confirmatory assays (A2/A3) run in parallel; no A1/A2 repetition	No
	Lao People's Democratic Republic	N/A	Not in compliance	Confirms HIV diagnosis with only 2 assays in low prevalence setting; For repeat discordant A1/A2, assays are repeated again instead of using an A3	Unclear
	Malaysia	2014	Not in compliance	Confirms HIV diagnosis with only 2 assays in low prevalence setting.; no mention of what to do for discordant results	No
	Nauru	2015	Not enough information	N/A	No
	Papua New Guinea	2010	Not in compliance	Confirms HIV+ diagnosis with only two assays in low prevalence setting	No
	Philippines	2010	Not in compliance	If A1 reactive, two lab-based tests run in parallel (PA and Ag-AB EIA); final test (WB) acts as tie-breaker, regardless of results of prior tests	No
	Singapore	2011	Not in compliance	No A3	No
	Vanuatu	2012	Not in compliance	Confirms HIV diagnosis with only 2 assays in low prevalence setting.; A2 and A3 run in parallel	No
	Viet Nam	2015	Not enough information	N/A	Unclear

Fonner VA, *et al. BMJ Global Health* 2020; 5:e001939. doi: 10.1136/bmjgh-2019-001939

b. Policies describing HIV testing in the context of PrEP

Region	Country	HTS follows national algorithm?	Description of PrEP testing strategy	Frequency of recommended testing
**AFRO**	Botswana	Not specified	Not specified	3 months
	Burundi	Not specified	Unknown	Not specified
	Democratic Republic of Congo	Not specified	Not specified	3 months
	Eritrea	Not specified	Not specified	Not specified
	Lesotho	Not specified	Patients with known HIV exposure within the past 72 hours should be offered PEP for 28 days. PrEP can be started after completing PEP if the patient remains HIV-negative.	3 months
	Namibia	Not specified	When HIV primary infection is suspected and/or when there is a history of possible recent HIV exposure; PrEP can be deferred for 4 weeks and the client re-tested to ascertain HIV status.	At PrEP initiation, 1 month, every 3 months
	Nigeria	Not specified	Not specified	3 months
	South Sudan	Not specified	When HIV primary infection is suspected and/or when there is a history of possible recent HIV exposure; PrEP can be deferred for 4 weeks and the client re-tested to ascertain HIV status.	3 months
	eSwatini	Not specified	Not specified	At initiation, then follow PrEP schedule (not specified)
	Uganda	Not specified	Not specified	3 months (1st time retest after 1 month)
	Zimbabwe	Not specified	1 rapid test to rule out HIV infection. If exam shows signs of acute infection, test for HIV RNA or antigen if available or defer PrEP for 4 weeks and retest then.	At initiation, every 3 months
	Kenya	Yes	HTS algorithm needs to be followed prior to PrEP initiation; mentions HIV test (singular) every three months	Rapid HIV antibody test every 3 months
	South Africa	Yes	Not specified	At one month, every 3 months
	Zambia	Yes	Not specified	3 months (1st time repeat after 28 days)
**EURO**	Belgium	Not specified	4th generation test	3 months (and at start of PrEP if high-risk and 1 month after initiation)

Fonner VA, *et al. BMJ Global Health* 2020; 5:e001939. doi: 10.1136/bmjgh-2019-001939

	France	Not specified	Not specified	1 month after start of treatment, then every 3 months
	Italy	Not specified	4th generation test	At initiation, 1 month visit, every 3 months after
	Netherlands	Not specified	4th generation Ag/Ab test; if acute infection suspected, order HIV RNA test	Before PrEP initiation, at PrEP initiation, 1 month after PrEP initiation, and then every 3 months
	Switzerland	Not specified	4th generation test	3 months
	European Union	N/A	4th generation test	3 months
**AMRO**	United States of America	No	4th generation Ag/Ab test; if acute infection suspected, order HIV RNA test; prohibits oral fluid	At initiation, every 3 months
	Barbados	Not specified	Not specified	Every 3 months; possibly after 1st month
	Canada	Not specified	4th generation test preferred; Additional NAT testing recommended if acute HIV infection suspected	3 months (1st time after 30 days)
	Brazil	Yes	Counter indicates use of RDT with oral fluid of acute HIV infection suspected	3 months (1st time within 2 weeks of 1st assessment)
**SEARO**	Myanmar	Not specified	Not specified	Before PrEP initiation and every 3 months
	Thailand	Unclear	Unclear	3 months (also after 1st month)
**WPRO**	Australia	Not specified	4th gen Ag/Ab test and assessment of primary HIV infection if suspected	3 months
	New Zealand	Not specified	4th gen Ag/Ab test and assessment of primary HIV infection if suspected	3 months

Fonner VA, *et al. BMJ Global Health* 2020; 5:e001939. doi: 10.1136/bmjgh-2019-001939
